# Oxidative stress induces dysregulated autophagy in corneal epithelium of keratoconus patients

**DOI:** 10.1371/journal.pone.0184628

**Published:** 2017-09-13

**Authors:** Rohit Shetty, Anupam Sharma, Natasha Pahuja, Priyanka Chevour, Neeraja Padmajan, Kamesh Dhamodaran, Chaitra Jayadev, Rudy M. M. A. Nuijts, Arkasubhra Ghosh, Jeyabalan Nallathambi

**Affiliations:** 1 Department of Cornea and Refractive surgery, Narayana Nethralaya Eye Hospital, Narayana Health City, Bommasandra, Bangalore, Karnataka, India; 2 GROW Research Laboratory, Narayana Nethralaya Foundation, Bangalore, Karnataka, India; 3 Maastricht University Medical Centre, Maastricht, The Netherlands; Univerzitet u Beogradu, SERBIA

## Abstract

Oxidative stress is one of the key factors that contributes to the pathogenesis of keratoconus (KC). Macroautophagy is a vital cellular mechanism that is activated in response to oxidative stress. The aim of this study was to understand the role of the autophagic lysosomal pathway in the oxidative damage of KC corneal epithelium and the human corneal epithelial cell line (HCE).The corneal epithelium was collected from 78 KC patients undergoing corneal cross-linking or topography guided photorefractive keratectomy. We performed microarray, qPCR and western blot analysis for the expression of autophagy markers on this epithelium from patients with different clinical grades of KC. A differential expression pattern of autophagy related markers was observed in the diseased corneal cone-specific epithelium compared to matched peripheral epithelium from KC patients with increasing clinical severity. Human corneal epithelial cells exposed to oxidative stress were analyzed for the expression of key proteins associated with KC pathogenesis and the autophagic pathway. Oxidative stress led to an increase in reactive oxygen species and an imbalanced expression of autophagy markers in the HCE cells. Further, reduced levels of Akt/p70S6 Kinase, which is a known target of the mTOR pathway was observed in HCE cells under oxidative stress as well as in KC epithelium. Our results suggest that an altered expression of proteins suggestive of defective autophagy and is a consequence of oxidative damage. This could play a possible role in the pathogenesis of KC.

## Introduction

Keratoconus (KC) is a common eye disease of the cornea associated with corneal thinning and irregular astigmatism, which results in progressive loss of vision [1, 2]. The estimated prevalence of this disease in the general population is approximately 1 per 2000 [2]. Current treatment options for severe and progressive cases include corneal transplantation and *in situ* corneal cross-linking, which are often associated with ocular morbidity and high costs. Non-surgical and non-invasive treatment modalities for the disease are not yet available, possibly due to a lack in the comprehensive understanding of the molecular basis of the disease. Oxidative stress is seen in several human diseases including KC [3, 4]. Solar ultraviolet radiations (UVR) are a source of oxidative stress to the cornea, rendering it susceptible to damage from free radicals and reactive oxygen species (ROS) [5]. The effect of oxidative damage and increased ROS has been observed in corneas of KC patients when compared to controls [6]. Tissue degradation due to oxidative damage was also found in KC corneal buttons when analyzing the expression levels of antioxidant enzymes [7, 8]. Environmentally-induced oxidative stress in KC causes an increase in ROS and decrease in the levels of antioxidants at a cellular level, which results in degradation of the extracellular matrix and subsequent thinning of the corneal stroma [3, 9]. Histopathological analysis of KC corneas shows thinning of the epithelium and the stroma within the apical cone region, Bowman’s layer breaks, focal fibrosis, and apoptosis of the anterior stromal keratocytes [10]. A cellular response to oxidative stress is the induction of macroautophagy (referred henceforth as autophagy) [11]. Autophagy is an evolutionarily conserved mechanism which causes degradation or clearance of long lived and misfolded proteins and damaged cellular organelles [12]. It is a protective mechanism against infections, cancer, neurodegeneration and aging [13]. Under normal cellular homeostasis, autophagy occurs at basal levels and is activated or up-regulated during oxidative damage. The mechanism of autophagy involves initiation of an autophagosome, which is termed as “induction”. This starts with the formation of a double membrane structure that prepares to engulf the material which is to be degraded [14]. The fusion of the autophagosome with hydrolase containing lysosomes to form autolysosomes is termed as autophagic flux [15]. These sequential steps are regulated by ubiquitin-like protein-conjugation systems and autophagy related proteins (ATGs) [16]. A failure of autophagy induction or autophagy flux (dynamics) due to oxidative stress can disturb the adaptive response and lead to autophagy mediated cell death [17, 18].

Although, genetic mechanisms, tear biomarkers [19] and inflammatory factors are involved in the pathogenesis of KC [20], the sequence of molecular events leading to progressive corneal ectasia remains unknown. To explore the possible role of autophagy in these events, we investigated the expression levels of autophagic lysosomal pathway related markers in KC patients’ corneal epithelium and human corneal epithelial (HCE) cells under oxidative stress. Our findings suggest that defective autophagic regulation may lead to detrimental consequences to the corneal epithelium as a result of oxidative damage, which could be a mechanism in the pathogenesis of KC.

## Materials and methods

### Study subjects, clinical evaluation and collection of corneal epithelium

This study was approved by the Narayana Nethralaya Ethics Committee/Narayana Nethralaya Institutional Review Board and followed the tenets of the Declaration of Helsinki. Both written and verbal consents were obtained from the patients for sample collection as per the ethical guidelines. All study subjects were clinically examined at the department of Cornea and Refractive Surgery, Narayana Nethralaya Eye Institute, Bangalore, India. Clinical examination included slit lamp examination with topographic and pachymetric evaluation on the Pentacam HR (Oculus, Germany) and Orbscan (Orbtek, Bausch&Lomb). Clinical grades of KC were determined by the Amsler-Krumeich classification and exclusion criteria for KC patients were followed as reported previously [21, 22]. Corneal epithelium from the central 8–9 mm of the cornea was debrided from patients and controls as described earlier [22, 23]. For the control epithelium (n = 18), subjects with normal corneal topography undergoing photorefractive keratectomy (PRK) for the correction of refractive errors were recruited. Corneal epithelium from KC patients (n = 60) undergoing corneal cross-linking (CXL) or topography guided photorefractive keratectomy (T-PRK) was collected. In controls undergoing PRK, after instillation of proparacaine eye drops, a pupil centered 4.5 mm diameter of cornea was trephine marked gently to ensure a superficial mark. The epithelium in this area was marked as the ‘centre’ and meticulously removed manually with a mechanical scraper whereas the surrounding epithelium extending approximately to 8.5 mm to 9 mm was considered as the ‘periphery’. Moist Merocel sponges were used to hydrate the underlying Bowman’s layer. Subsequently, excimer laser was performed for the refractive error correction. In KC subjects undergoing CXL or T-PRK, the location of the cone was determined by corneal topography. A 4.5 mm diameter of the cornea was trephine marked and centered on the ‘cone’ apex. The epithelium overlying this area and the epithelium over the peripheral cornea were separately scraped with a mechanical scraper. The derived corneal epithelium was immediately placed on dry ice until transfer to −80°C for storage. Study subjects and clinical characteristics are shown in Table 1.

**Table 1:** Clinical observations of keratoconus patients.

Clinical details	Control	Grade 1	Grade 2	Grade 3	P value
**Age**	22.4±2.441	20.47±1.527	26.08±1.916	20±2.542	0.1021
**K1**	42.8±0.7321	43.29±0.3152	48.42±0.3624	49.1±1.677	<0.001
**K2**	43.78±0.9687	47.18±0.6221	52.71±0.6173	54.21±1.646	<0.001
**KM**	43.28±0.8429	45.13±0.4099	50.45±0.3719	51.49±1.516	<0.001
**Sphere**	-1.85±0.341	-0.1333±0.4329	-2.521±0.55	-3.714±1.74	0.0058
**Cylinder**	-0.45±0.2784	-2.35±0.337	-2.042±0.6	-2.714±0.55	0.0058

This table describes the pachymetry data from Pentacam and refraction measurements on corneal thickness. Sphere and Cylinder: Spherical and Cylindrical refraction values. K1, K2: Readings of corneal curvature by keratometry. Km and K-Max: Mean and maximum keratometry value. The ANOVA p value column shows group statistics.

### Oxidative stress in cell culture

Human corneal epithelial (HCE) and human corneal stromal fibroblast cells (HCF) were cultured at a concentration of 0.3x10^6^ cells/ml in a medium (Hams F-12 DMEM, Gibco, USA) containing 10% fetal bovine serum (Gibco,USA), 100 U/mL penicillin, and 100 mg/mL streptomycin sulphate (Sigma-Aldrich, St. Louis, MO) at 37°C. To induce oxidative stress, HCE cells were exposed to hyperoxic conditions for 3 and 7 days at 40% O_2_ and 5% CO_2_ using a multi gas incubator (SMA-80DS/165, ASTEC, Japan). For normoxic conditions, cells were incubated under a normal physiological state of 21% O_2_ and 5% CO_2_. Trypanblue staining was used to check the viability of cells under hyperoxic conditions treated with or without chloroquine (30nM, Sigma Aldrich), trehalose (100mM, MP Biomedicals) and rapamycin (500nM, Calbiochem).

### RNA isolation, microarray and quantitative real-time PCR

Total ribonucleic acid (RNA) extraction was performed on epithelial tissues and HCE cells by using the RNeasy Mini Kit (Qiagen) according to the manufacturer’s protocol and quantified using a nanodrop spectrophotometer (NanoDrop 1000, Thermo Scientific, DE, USA). Microarray and data analyses followed the protocols as described previously [24]. 1μg of the total RNA was converted to cDNA using the Biorad iScript™ cDNA Synthesis Kit. Quantitative real-time PCR was performed using 4μl of 10-fold diluted cDNA in a final volume of 10μl using the SYBR Green master mix 2X (Bio-Rad, Philadelphia, PA, USA) according to the manufacturer’s instruction. PCR primer sequences used for the gene expression analysis are mentioned in Table 2. βactin was used as an internal control of mRNA expression analysis.

**Table 2:** Primers used for qRT- PCR analysis.

Gene name	Sequences (5’-3’)	Gene acc no
***COL IVA1***	FP: GCAAACGCTTACAGCTTTTGG RP: GGACGGCGTAGGCTTCTTG	NM_001845
***TIMP1***	FP: TGTTGTTGCTGTGGCTGATA RP: CTGATGACGAGGTCGGAATTG	NM_003254
***MMP9***	FP: GGGCTTAGATCATTCCTCAGTG RP: GCCATTCACGTCGTCCTTAT	NM_004994
***LOX***	FP: ACATTCGCTACACAGGACATC RP: TTCCCACTTCAGAACACCAG	NM_002317
***IL6***	FP: GATGAGTACAAAAGTCCTGATCCA RP: CTGCAGCCACTGGTTCTGT	NM_54894
***LC3A***	FP: CGTCCTGGACAAGACCAAGT RP: CTCGTCTTTCTCCTGCTCGT	NM_032514
***LC3B***	FP: AGCAGCATCCAACCAAAATC RP:CTGTGTCCGTTCACCAACAG	NM_022818
***LAMP1***	FP: AGTGGCCCTAAGAACATGACC RP: AGTGTATGTCCTCTTCCAAAAGC	NM_005561
***ATG4***	FP: GGAACAACGTCAAGTACGGTT RP: CTCGCCCTCGAAACGGTAG	NM_032885
***ATG5***	FP: AAAGATGTGCTTCGAGATGTGT RP: CACTTTGTCAGTTACCAACGTCA	NM_004849
***β ACTIN***	FP: GCCAACCGCGAGAAGATGA RP: CCATCACGATGCCAGTGGTA	NM_001101

### Protein lysate preparation and western blot analysis

HCE cells and the corneal epithelial tissues were lysed on ice using radio-immunoprecipitation (RIPA) assay buffer (Cat-786-489, G-Biosciences, USA) containing protease and phosphatase inhibitors (Roche life science, USA). The cells were then snap frozen and thawed 3 times followed by centrifugation at 13000 rpm for 10 minutes at 4°C. Protein concentration was measured using the bicinchoninic acid (BCA, G-Biosciences, USA) assay. Equal amounts of protein (20μg) were loaded and run on 10% SDS PAGE, blotted onto PVDF membrane, blocked with 5% non-fat dry milk powder in PBST followed by overnight incubation with antibodies of LAMP1 [1:1000, Cell Signaling (D2D11) XP], LC3A/B [1:1000, Cell Signaling, LC3A/B #4108], ATG5 [1:1000, Cell Signaling (D1G9)], ATG12 [1:1000, Cell Signaling Atg12 (D88H11)], SQSTM1/p62 [1:1000, Cell Signaling #5114], p70 S6 Kinase [1:1000, Cell Signaling, (9202)], phospho70S6 Kinase T389 [1:1000, Cell Signaling,(9205)], Akt [1:1000, Cell Signaling, (4691)], phosphoAktS473 [1:1000, Cell Signaling,(9271)], MMP9 [1:500, Santa Cruz, SC-13520], LOX [1:500, Merck Millipore, ABT-112], Collagen IV [1:1000, Abcam, (ab6586)], β-actin [1:3000, Santa Cruz, C-4] and GAPDH [1:500, Abgenex, Clone: ABM22C5]. The secondary antibodies (anti-rabbit, anti-mouse) were conjugated with horseradish peroxidase and a chemiluminescence substrate (Biorad, Philadelphia, PA, USA) was used to visualize the band (ImageQuant LAS 500, GE Healthcare Life Sciences, USA).

### Flow cytometry analysis

Experiments were performed in BD FACS Calibur^TM^. The HCE cells were trypsinized and 10,000 cells were recorded for analysis under both normoxic and oxidatively stressed conditions on Day 3 and 7. Quantification of ROS (2’,7’–dichlorofluorescin diacetate [DCFDA], Life Technologies, FL-1), autophagosomes (CYTO-ID Autophagy Detection Kit, Enzo Lifesciences, FL-1) and lysosomal content (LysoTracker® Red DND-99, Life Technologies, FL-3) were assessed as per the manufacture’s protocol. Data was analyzed with the Cell Quest Pro Software (Version 6.0).

### Fluorescence imaging

For imaging, the HCE cells were plated on 8-chamber slides (SPL Life Sciences) at a density of 100 cells/chamber and subjected to normoxic and oxidative stress conditions until Day 7. For monitoring autophagosomes, HCE cells were incubated with CYTO-ID dye (2μl of CYTO-ID in 1ml of 1X assay buffer) for 30 min. Lysosomal content was monitored using LysoTracker® Red DND-99 (500nM) for 15 mins at 37°C (Life Technologies, MA, USA). Cells were fixed with 4% paraformaldehyde (pH7.2), washed and examined under fluorescent microscope (Zeiss, Oberkochen, Germany) and the images were captured using Axiocam, a ZEN software. Numbers of cells counted for red and yellow fluorescence were more than 25 for each condition.

### Transfection

HCE cells were grown on glass coverslips in 6-well culture plates. 3μg of LC3-eGFP-mRFP (ptfLC3) [Addgene, Plasmid #21074] were transfected using the lipofectamine LTX Plus reagent (Thermo Fischer Scientific, MA, USA) according to the manufacturer’s instructions. After 24 hrs of transfection, cells were exposed to normoxic and hyperoxic conditions. Cells were examined under the fluorescence microscope (Zeiss, Oberkochen, Germany) for the expression of GFP, mRFP proteins and co-localization.

### Statistical analysis

All experiments were performed at least three independent (n = 3) times except for the microarray analysis. The statistical significance of the differences was analyzed by one-way analysis of variance (ANOVA) and student *t* test using GraphPad software. Asterisks *, **, and *** denote a significance with p-Values <0.05, 0.01, and 0.001 respectively.

## Results

### Expression of autophagy pathway related genes in the corneal epithelium of KC patients

**Fig 1:**
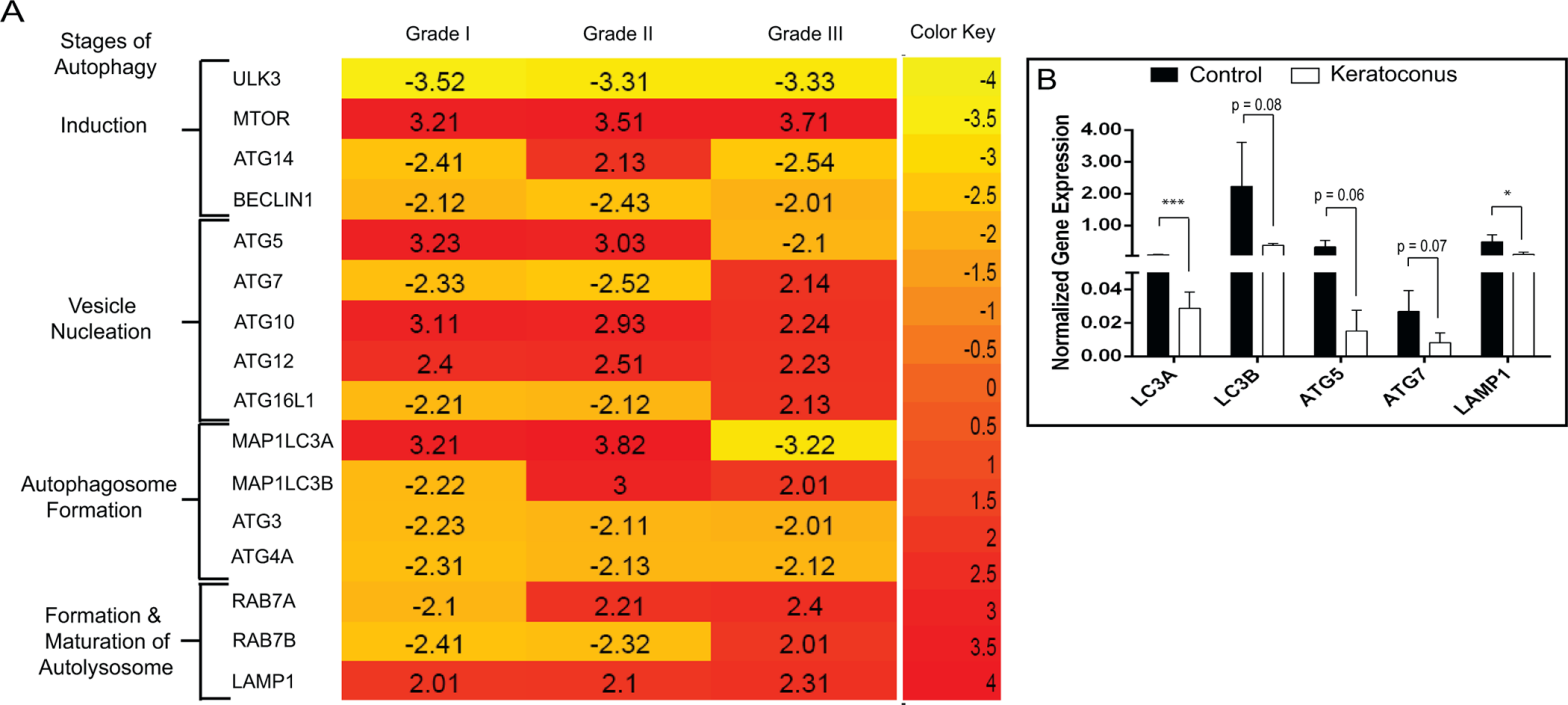
mRNA expression analysis of autophagy pathway related genes in whole corneal epithelium from KC patients. (A) Differential expression pattern of autophagy related genes in clinical grades (I, II, III) of Keratoconus (KC) epithelium. Genes up and down-regulated shows the cut-off fold difference of 1.5 (n = 1) (B) Gene expression profile (LC3A, LC3B, ATG5, LAMP-1) of pooled clinical grades of KC epithelium. qPCR results were normalized with β-actin. Data are the means ± SD, n = 3, statistical significance was denoted (*p < 0.05, *** p < 0.001, compared to the mRNA expression levels of control epithelium).

Using microarray, the expression of autophagy pathway associated genes was compared between whole epithelium of different clinical grades of KC (n = 5 of each grade) and non-ectatic controls (n = 5). This analysis revealed a differential expression pattern of genes (increased or decreased fold change in expression) involved in the various stages of the autophagic lysosomal degradation pathway (Fig 1A) GEO accession number: GSE92935. Further validation of the mRNA transcript levels of LC3A, LC3B, ATG5, ATG7 and LAMP1 showed a lower expression of KC epithelium in pooled clinical grades of KC (n = 3 of each grades) when compared to the control epithelium (n = 3) (Fig 1B).

#### Dysregulated expression of autophagy markers in cone versus periphery of KC corneal epithelium

**Fig 2:**
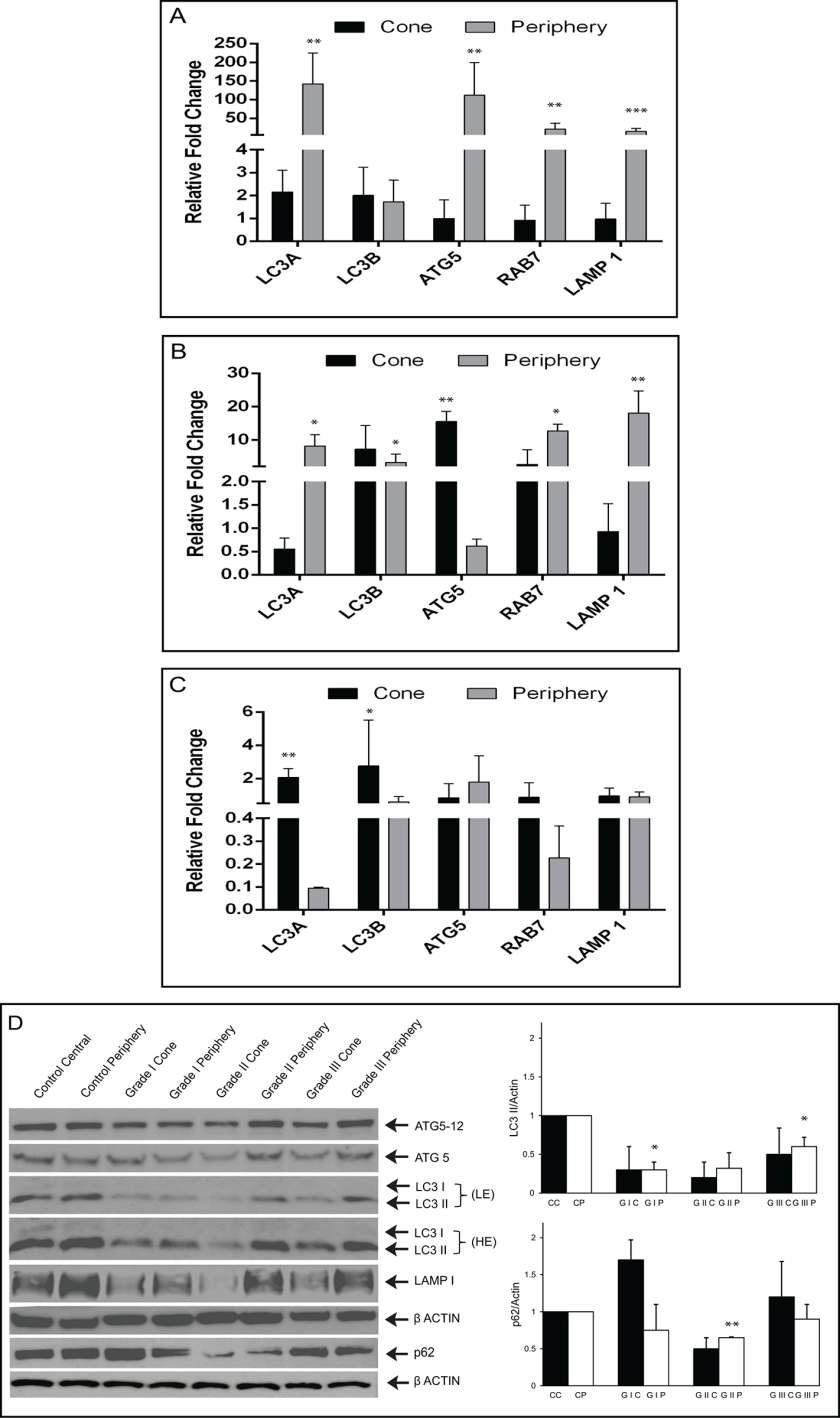
Differential expression pattern of autophagy related markers in the cone and peripheral region of KC corneas. mRNA expression levels of LC3A, LC3B, ATG5, ATG7, RAB7 and LAMP1 (A) Clinical grade I (B) Clinical grade II (C) Clinical grade III. Data are the means ± SD, n = 3, statistical significance was denoted as (*** p < 0.001, **p < 0.01, *p < 0.05 compared to central and peripheral regions of control epithelium). (D) Immunoblot shows the protein levels of LC3, ATG5, p62 and LAMP1 across KC corneas with clinical grades (cone and peripheral regions). Densitometric analysis of the blots showing the ratios of LC3-II and p62 to β-actin. **CC**- Control central, **CP**-Control periphery, **GIC**-Grade I Cone, **GIP**-Grade I periphery, **GIIC**-Grade II Cone, **GIP**-Grade II periphery, **GIIIC**-Grade III Cone, **GIP**-Grade III periphery. Data are the mean ± SD values, n = 3, statistical significance was denoted **p < 0.01, *p < 0.05 compared to levels of control (central and peripheral regions) epithelium.

The KC cornea is characterized by localized corneal thinning and protrusion due to a loss of corneal stiffness (the ectatic cone area) surrounded by an area of normal thickness (non-ectatic zone). To determine whether autophagy deregulation is only restricted to the diseased or ‘cone’ area, we obtained epithelium separately from the cone and the peripheral cornea of KC patients (total KC epithelium-36, n = 12 of each clinical grade, control epithelium n = 10). We then investigated the expression of autophagy pathway related genes LC3A, LC3B, ATG5, ATG7, RAB7 and LAMP1 in clinical grades (I to III) of KC patients’ epithelium. The mRNA transcript and protein level expressions of the cone (ectatic) and peripheral (non-ectatic) regions in KC were compared with control corneal epithelium. The epithelium from the peripheral region of clinical grades I and II showed an increased mRNA level of LC3A, RAB7 and LAMP1 when compared to the cone region epithelium (Fig 2A and 2B) of the same patients. Comparison of the cone and peripheral area of KC Grade III epithelium revealed no difference in the mRNA expression profile of LC3A, RAB7 and LAMP1 (Fig 2C). In comparison to the controls, corneal epithelium from different clinical grades of KC patients showed decreased levels of LC3-II and LAMP1 protein in the cone and peripheral regions (Fig 2D). There was no significant difference in the protein levels of ATG5 and ATG12. When compared to the control epithelium, there was an increased level of p62 protein in the cone and decrease in the peripheral regions of KC Grades I and III. Additionally, KC Grade II epithelium showed decreased levels of p62 in the cone and peripheral regions (Fig 2D).

### Hyperoxia as an experimental condition to induce oxidative stress in human corneal epithelial cells

**Fig 3:**
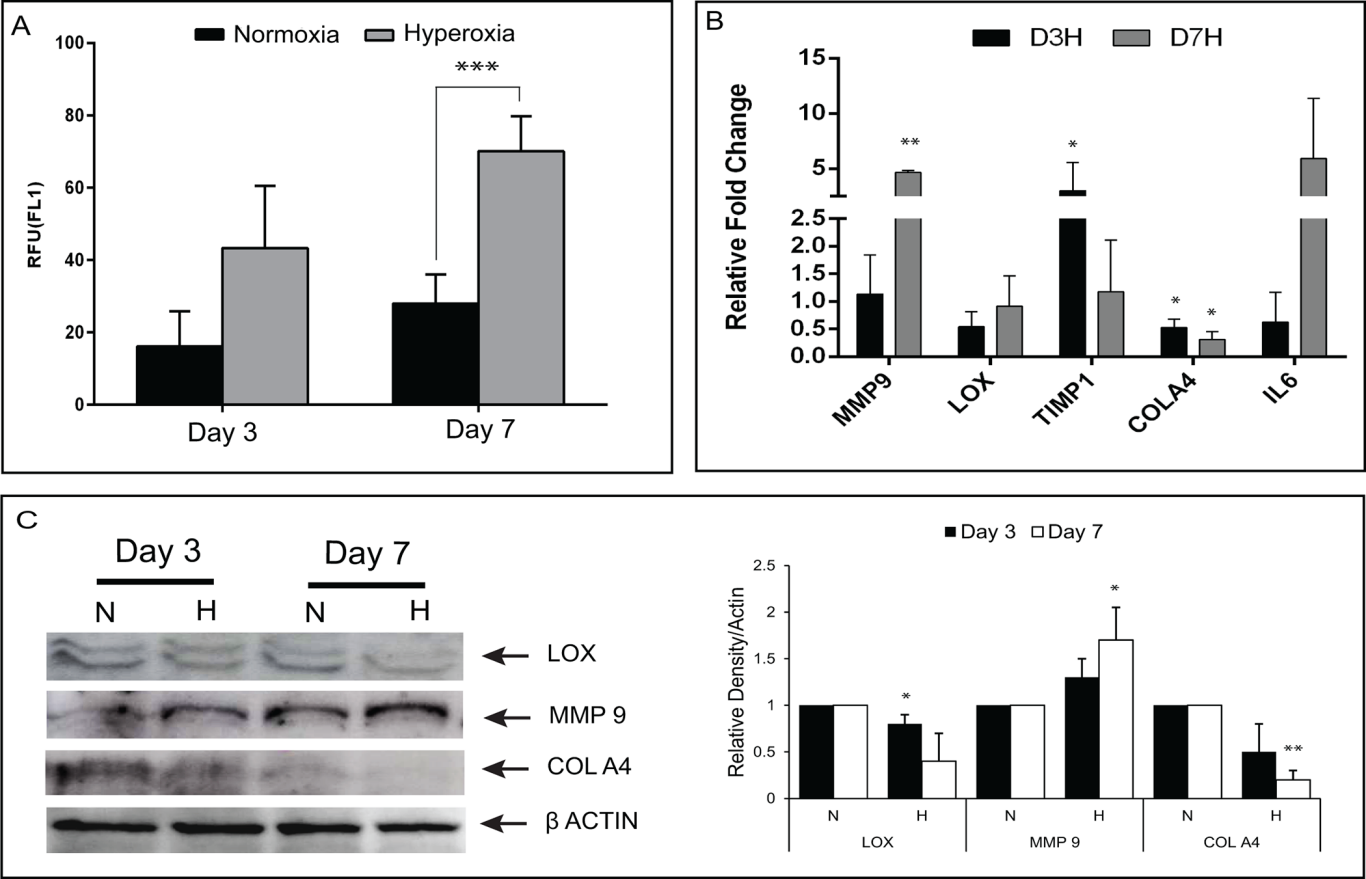
Expression of KC associated markers in human corneal epithelial cells under oxidative stress at 3 and 7 days. HCE cells were incubated under normoxic (N) (21% O_2_) and hyperoxic (H) (40% O_2_) conditions. (A) Intracellular ROS production represented graphically in percentage and measured by flow cytometry using DCFDA. Data are the mean ± SD values, n = 3, significant difference was denoted *** p < 0.001 compared to cells in normoxic conditions. (B) Quantitative PCR analysis of genes MMP9, IL6, COLIVA1, LOX, and TIMP1. qPCR results were normalized with β-actin. Data are the means ± SD, n = 3, significant difference was denoted as (**p < 0.01, *p < 0.05 compared to cells in normoxia at day 3 and day 7). (C) Western blot and densitometric analysis of MMP9, COLIVA1 and LOX proteins with β-actin used as a loading control. Densitometry data are the mean ± SD values, n = 3, statistical significance denoted as (**p < 0.01,*p < 0.05 compared to cells incubated in normoxic conditions at day 3, day 7).

HCE cells exposed to hyperoxic conditions (40% O_2,_ for 3 and 7 days) were assessed for viability (more than 92%) using trypan blue staining (data not shown). These cells demonstrated a significant increase in the levels of reactive oxygen species (ROS) compared to normoxic cells (21% O_2_) (Fig 3A). We tested KC associated gene markers [22, 25] (MMP9, IL6, COLIVA1, LOX, and TIMP1) under hyperoxic conditions. No difference was observed in the mRNA expression level at Day 3, but cells cultured until Day 7 showed an increase in the levels of MMP9 and IL6 and decrease in levels of TIMP1 and COLIVA1 (Fig 3B). The expression levels or fold change of MMP9, TIMP1 and IL6 transcripts were significant when compared to normoxic exposure. In oxidative stress conditions, the western blot analysis indicated decreased levels of COLIVA1 and LOX with elevated levels of MMP9 when compared to normoxic exposure of HCE cells (Fig 3C).

### Expression of autophagy markers in HCE cells exposed to oxidative stress

**Fig 4:**
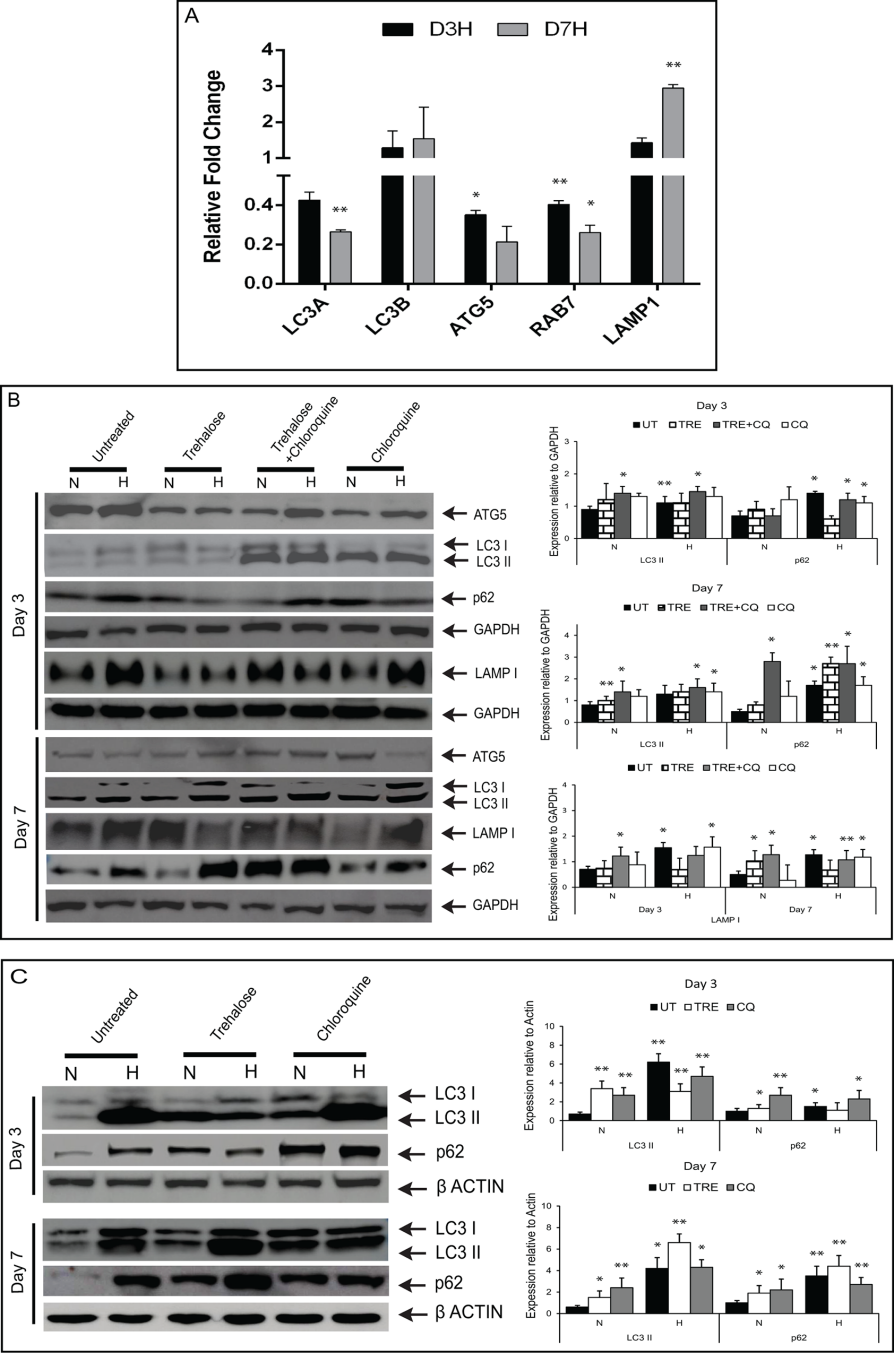
Analysis of autophagy markers in HCE cells exposed to oxidative stress. (A) HCE cells exposed to normoxic (N) (21% O_2_) and hyperoxic (H) (40% O_2_) conditions at day 3 and day 7. Quantitative PCR analysis of genes LC3A, LC3B, ATG5, RAB7 and LAMP1 were normalized with β-actin. Data are the mean ± SD values, n = 3, significant difference was denoted as **p < 0.01, *p < 0.05 compared to cells in normoxia. (B) HCE cells exposed to normoxic (N) (21% O_2_) and hyperoxic (H) (40% O_2_) conditions treated with trehalose, choloroquine or combination of both at day 3 and day 7. Western blot results (LC3, ATG5, p62 and LAMP-1), Densitometry data are the mean ± SD values, n = 3, statistical significance denoted as **p < 0.01, *p < 0.05. All treatments (trehalose and/or chloroquine) under normoxic and hyperoxic conditions were compared to untreated normoxic cells. (C) HCF (human corneal fibroblast) cells under normoxic (N) (21% O_2_) and hyperoxic (H) (40% O_2_) conditions with and without trehalose (24hrs), chloroquine (4hrs) treatment at day 3 and day 7. Immunoblot and densitometric analysis showing LC3-II, P62 levels relative to β-actin. Densitometry data are the mean ± SD values, n = 3, statistical significance denoted as **p < 0.01, *p < 0.05. All treatments (trehalose or chloroquine) under normoxic and hyperoxic conditions were compared to untreated normoxic cells.

The analysis of autophagy related markers (LC3A, LC3B, ATG5, RAB7 and LAMP-1) revealed a significant increase in mRNA (Fig 4A) levels of LC3B (autophagosome marker) and LAMP1 (lysosomal associated membrane protein) (Fig 4B) in HCE cells exposed to oxidative stress on Day 3 and 7. LC3 and LAMP1 proteins turnover were assessed by treating the cells with trehalose (autophagy inducer for 24 hrs) and chloroquine (lysosomotropic agent that prevents fusion of endosomes and lysosomes for 4 hrs). Compared to normoxic conditions on Day 3 and 7, HCE cells exposed to hyperoxic condition showed increased proteins levels of LC3-II, LAMP1 and p62 (Fig 4B). In the presence of chloroquine alone or in combination with trehalose, oxidatively stressed HCE cells showed no significant difference in the levels of LC3-II and LAMP1 compared to untreated conditions (Fig 4B). In comparison with untreated conditions, the cells treated with trehalose and chloroquine showed no increase in p62 on Day 3. Additionally, in HCE cells treated with chloroquine alone, there was an increased p62 protein level in on Day 7 and no change in Day 3 compared with untreated conditions (Fig 4B). Autophagy is a dynamic process and varies substantially between different tissues and cell types. Hence, we measured LC3-II turnover of HCF (human corneal stromal fibroblast) cells under hyperoxic conditions, with or without treatment of trehalose and chloroquine. The results indicate an increased protein levels of LC3-II and p62 in cells under hyperoxic exposures compared to normoxic conditions on Day 3 and Day 7 (Fig 4C). With the addition of chloroquine there were no significant changes in LC3-II and p62 levels in HCF cells under hyperoxia compared to untreated conditions.

### Quantification of autophagosomes and autolysosomes in HCE cells under oxidative stress

**Fig 5:**
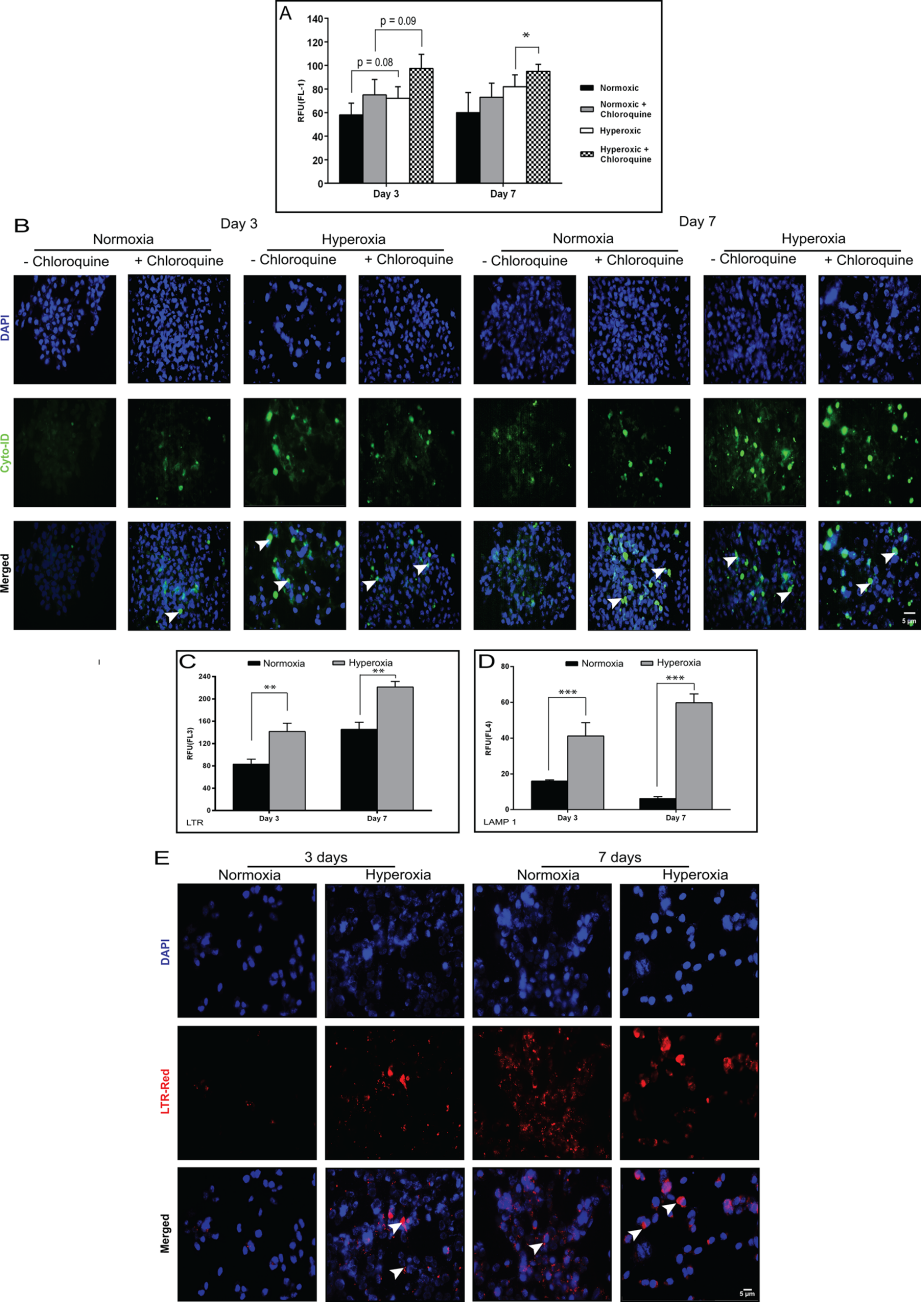
Measurement of autophagosomes and autolysosomes in HCE cells under oxidative stress. (A, B) HCE cells exposed to normoxic (21% O_2_) and hyperoxic (40% O_2_) conditions with and without chloroquine (4hrs) treatment at day3 and day7. (A) Quantification of autophagosomes using CytoID staining by flow cytometry in FL-1 channel. CytoID positive cells are represented graphically. Data are the mean ± SD values, n = 3, significant difference was denoted as *p < 0.05 compared to cells in normoxia with or without chloroquine treatment at 3 and 7 days. (B) Fluorescence imaging analysis of CytoID [white arrow indicates green fluorescence/autophagosomes in the peri nuclear region]. (C, D, E) HCE cells exposed to normoxic (21% O_2_) and hyperoxic (40% O_2_) conditions. (C, D) Lysosomal content analyzed by flow cytometry using LysoTracker Red-FL3 and LAMP1-APC (CD107a) (FL-4). Data are the mean ± SD values, n = 3, significant difference was denoted *** p < 0.001, **p < 0.01, compared to cells in normoxic condition at 3 and 7 days. (E) HCE cells show perinuclear red staining (LysoTracker Red) of the lysosomal mass under fluorescence image analysis (white arrows). Scale bar: 5μm

Quantification of autophagosomes was done by flow cytometry using the Cyto-ID^TM^ autophagy detection kit. The analysis shows that the autophagosomes (Cyto-ID fluorescence) had increased in number on Day 3 and 7 in oxidatively stressed cells with or without chloroquine treatment (Fig 5A). These results were further confirmed by the accumulation of green dye in the perinuclear region (localization of autophagosomes) of the HCE cells by fluorescence imaging analysis (Fig 5B). We used LysoTracker Red (LTR), fluorescence dye and LAMP-1 APC (CD107a) to check the level of lysosomal content in HCE cells under oxidative stress. There was a significant increase in the lysosomal mass in corneal cells under oxidative stress compared to untreated cells (Fig 5C and 5D). To confirm further, oxidatively stressed cells stained with LTR and analyzed under fluorescence microscopy demonstrated an increase of red fluorescence in the cytoplasmic region on Day 3 and 7 (Fig 5E).

### Changes in autophagy dynamics in HCE cells exposed to oxidative stress

**Fig 6:**
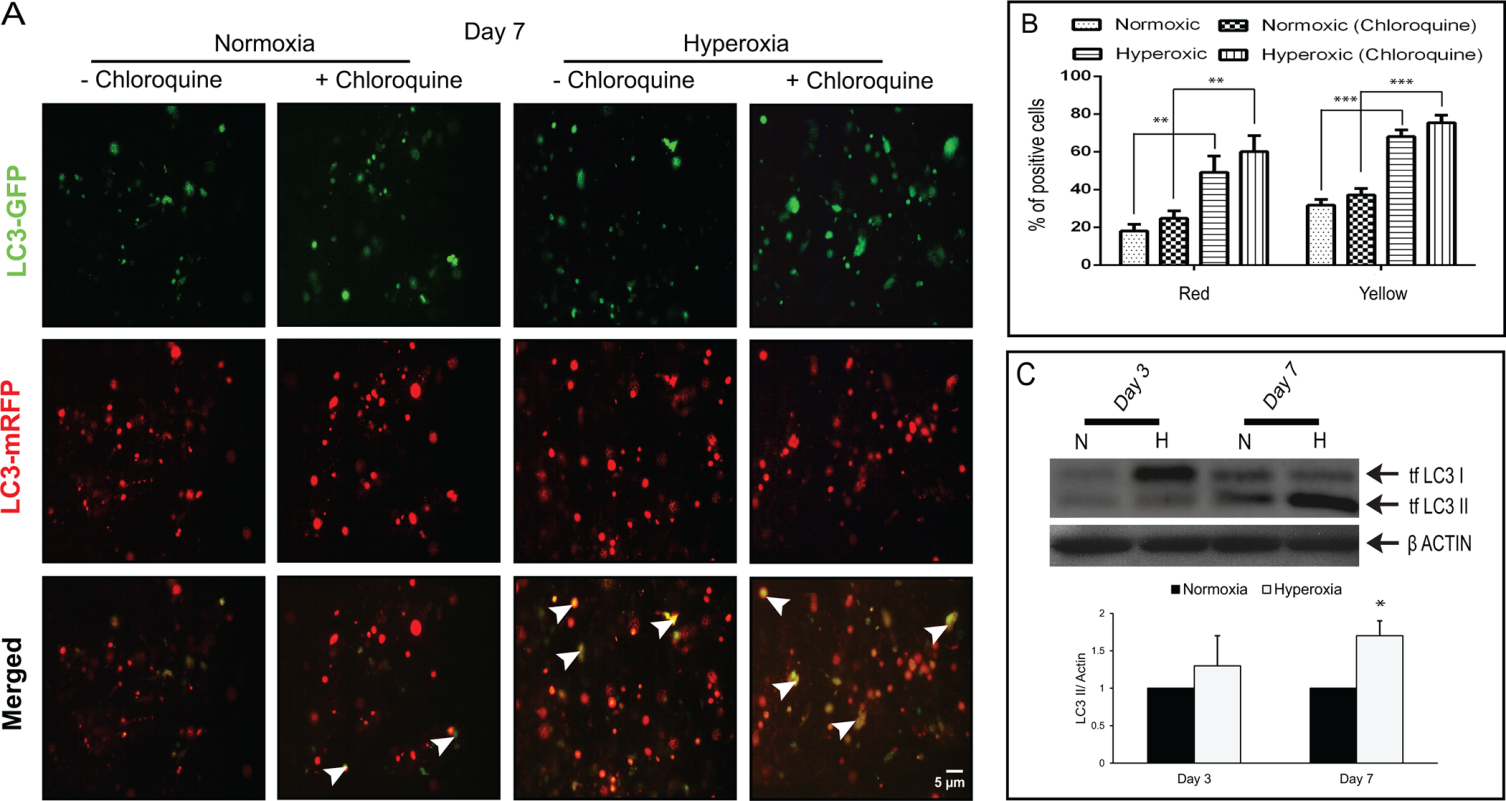
Autophagy dynamics in oxidatively stressed HCE cells. Monitoring autophagy dynamics in oxidatively damaged HCE cells treated with or without chloroquine with transfection of tfLC3 plasmid (A) Fluorescence image and expression of LC3 tagged with GFP and mRFP fluorescent proteins. The arrows indicate the co-localization of GFP/mRFP (yellow fluorescence); scale bar, 5μm. (B) Percentage of positive yellow and red fluorescence cells are represented graphically. Data are the mean ± SD values, n = 3, statistical significance was calculated based on comparison of visual scoring of three independent observers and denoted as *** p < 0.001, **p < 0.01 compared to cells in normoxia with or without chloroquine treatment at day 7. (C) Protein expression level of tfLC3 detected by western blot on Day 3 and 7 with densitometric analysis relative to β-actin. Densitometry data are the mean ± SD values, n = 3, statistical significance was denoted as (*p < 0.05 compared to normoxic condition)

To assess autophagy dynamics in HCE cells in oxidative stress conditions, we transfected tfLC3 plasmid (LC3 tagged with eGFP and mRFP proteins) into HCE cells. On Day 7, compared to normoxic condition, oxidatively stressed cellstreated with and without chloroquine showed an increase in yellow (co-localization of eGFP/mRFP autophagosomes or autolysosomes) and red fluorescence on fluorescence image analysis (Fig 6A). In addition, oxidatively stressed HCE cells displayed a higher percentage of positive red and yellow fluorescence compared to normoxic cultures (Fig 6B). These findings were confirmed with western blot analysis, which demonstrated higher protein levels of LC3-I and LC3-II in cells cultured under a 40% O_2_ on Day 3 and 7 day (Fig 6C).

### Changes in Akt/p70S6 kinase phosphorylation in keratoconic epithelium and HCE cells under oxidative stress

**Fig 7:**
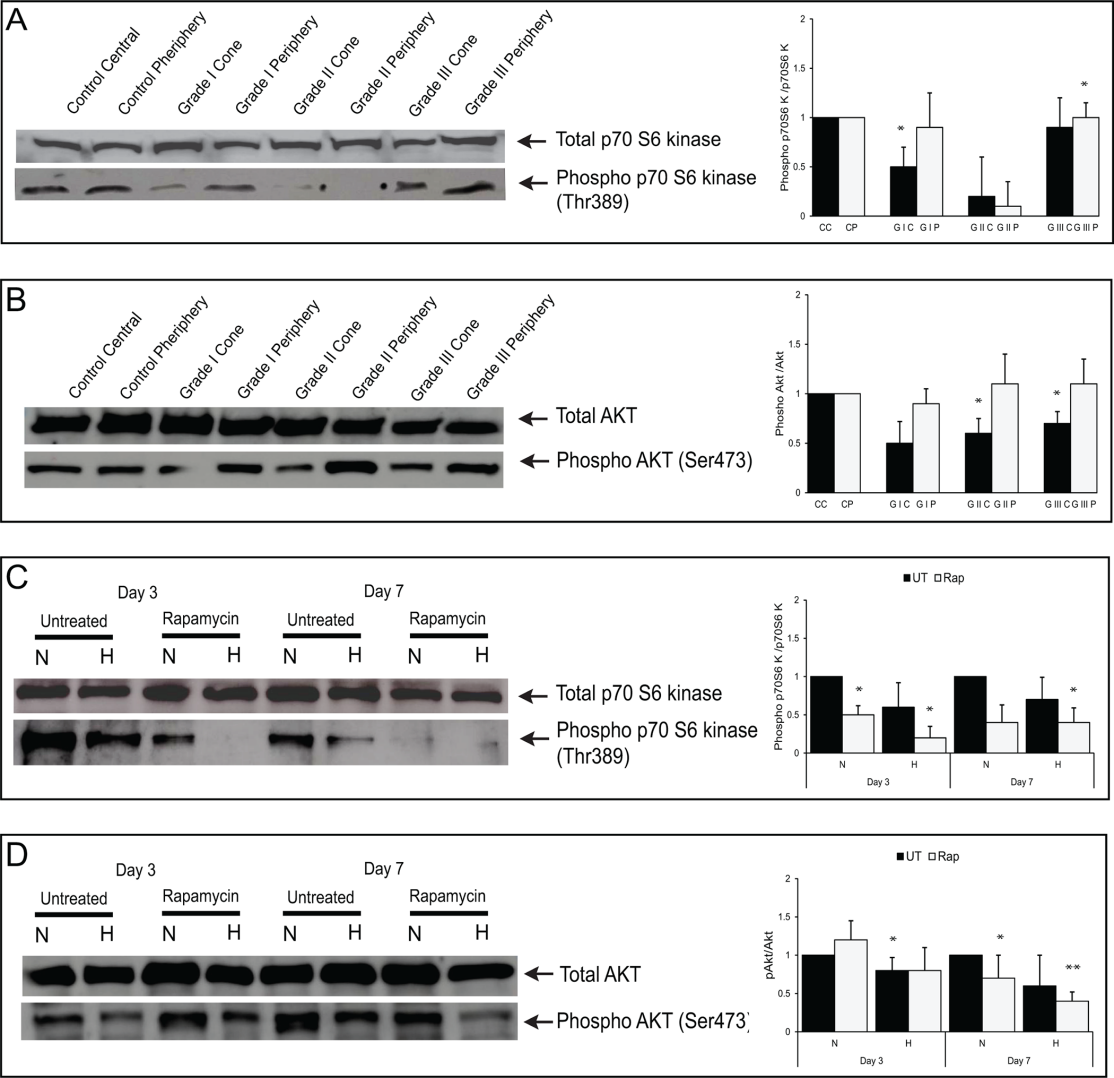
Akt /p70S6 kinase phosphorylation status in the keratoconic epithelium and HCE cells under oxidative stress. Expression levels of total and phosphorylated Akt (Ser473/p70s6kinase (Thr389) were analyzed by western blot and densitometric methods. (A, B) Cone and peripheral regions of KC corneas with clinical grades I to III. Densitometry data are the mean ± SD values, n = 3, statistical significance denoted as (*p < 0.05 compared to levels of total p70S6 kinase, Akt in control epithelium). (C, D) HCE cells exposed to normoxic (21% O_2_) and hyperoxic (40% O_2_) conditions with or without rapamycin treatment on Days 3 and 7. Densitometry data are the mean ± SD values, n = 3, statistical significance denoted as (**p < 0.01,*p < 0.05 compared to levels of total p70S6 kinase and Akt in cells exposed to normoxic conditions).

Decreased phosphorylation levels of phosphoAkt (Ser473)/p70S6 Kinase (Thr389) were observed in HCE cells under oxidative stress as well as in the cone region of KC corneal epithelium compared to normoxic cells and control epithelium (Fig 7A–7D).

## Discussion

In this study, we explored the oxidative damage to the cornea and its correlation with the autophagic lysosomal pathway in the pathogenesis of KC. It is known that when the cornea is exposed to a variety of environmental stressors including oxidative stress, it produces increased levels of free radicals (ROS), which can lead to corneal damage and dysfunction [5, 6]. The accumulation of MDA (malondialdehyde for lipid peroxidation) and NT (3-nitrotyrosine for peroxynitrite formation) in KC corneal epithelium and stroma suggests that free radicals/ROS have a major role in the pathogenesis of the disease [7]. Furthermore, KC corneas have shown increased accumulation of superoxides, hydrogen peroxide and hydroxyl radicals with decreased levels of antioxidant defenses [8, 26, 27]. On investigating the differential expression profile, LC3-II and LAMP-1 proteins (cone and periphery) were reduced across all clinical grades of KC epithelium, compared to controls. In addition, we found LC3-II and LAMP-1 were significantly decreased in the cone region of KC epithelium from Grades II and III compared to the peripheral region. This could be due to insufficient autophagy activity in the diseased area (cone) compared to the matched peripheral region during disease progression or an altered expression of autophagic markers, which has been observed in other disease conditions [28, 29]. A difference in the expression levels (low, medium, high) of autophagosomal marker LC3 has been shown in KC epithelium, but without clinical disease classification, thereby demonstrating alterations in the autophagic pathway [30]. Similarly, distorted expression levels of hepatocyte growth factor (HGF) and its receptor mesenchymal-epithelial transition factor (c-Met/Met) have been found in the peripheral and cone regions of KC epithelium [31]. In KC Grade III, we found a slight increase in LC3 and LAMP1 proteins compared to other clinical grades (I,II) suggesting that accumulation of autophagosome and lysosome or impaired fusion may be responsible for the abnormal dynamics of autophagy in the diseased areas of the cornea. We observed reduced differences in gene expression levels of Grade III samples between the cone and periphery. This may be attributed to the disease being more severe and therefore affecting a wider area that encompasses the peripheral regions of the KC cornea, which is not evident at the phenotypic level of clinical evaluation. We have made similar observations in our earlier study on cone vs peripheral epithelial gene expression alterations in KC [23].

A previous study reported the differential expression profiles of LC3 and Secreted Frizzled-Related Protein 1(SFRP1) in KC epithelium suggesting crosstalk between Wnt signaling and autophagy [30]. We hypothesize that accumulation of excessive non-degraded material in the lysosomal compartment deregulates its function, which requires further investigation in the KC cornea. Moreover, elevated levels of lysosomal enzymes like cathepsins B, G and V/L were found in KC corneas [3, 32]. Interestingly, the high level of autofluorescence observed in KC corneal epithelium might be a consequence of increased lipofuscin deposits or non-degradable cellular components in the lysosomal compartment [30]. It has been reported that oxidative stress might be involved in the damage of lysosomal membranes, causing the release of proteolytic enzymes and triggering corneal thinning [3]. It has been noted that different levels of LAMP1 proteins in KC epithelium (between the cone and periphery) among the clinical grades when compared to controls, suggesting the involvement of lysosomal function in early and late stages of the disease. p62, also called as SQSTM1/sequestome 1, has a major role in the association of LC3 and ubiquitinated substrates, which is degraded by the autophagic machinery [33, 34]. We observed increased p62 levels in cone regions of clinical Grades I and III but decreased levels in peripheral regions across all grades when compared to controls. These observations suggest the possibility of suppression of autophagy in the diseased area (cone) compared to the non-diseased. It is possible that transcription factor EB (TFEB) mediated activity may be involved in KC corneas, since its transcriptional activation has been demonstrated during lysosomal biogenesis in response to oxidative stress [35].

To simulate oxidative damage and investigate the molecular mechanisms in KC, HCE cells were exposed to a hyperoxic environment, which has the advantage of very low toxicity or cell death and has been previously used in experimental conditions [36, 37]. One of the known markers for oxidative damage, intracellular ROS, was increased in cells exposed to 40% O_2_ which has been shown in KC corneas as well [6]. In addition, products of mtDNA damage was found in KC corneas, which indicates that increased oxidative stress is important in the pathogenesis of KC [38]. Under oxidative stress conditions in HCE cells, we observed up-regulated mRNA and protein levels of MMP9 and IL-6 with down-regulated TIMP1, COLIVA1 and LOX which reiterates our previous findings in KC patients’ epithelium [3, 22, 25]. Increased mRNA expression and proteins levels of LC3 and LAMP1 in oxidatively stressed cells might lead to induction of autophagy, a potential cellular response to oxidative damage [11, 35, 39] or increased levels of ROS in HCE cells.

Western blot analysis showed increased levels of LC3-II in oxidatively stressed HCE cells compared to normoxic conditions. This increase in the level of LC3-II implies either an induction of autophagy or blockage of autophagosomal maturation/degradation [40]. To determine whether the increase in LC3-II levels in oxidatively stressed cells is due to an induction of autophagy or decreased autophagic flux, cells were treated with chloroquine. In the presence of chloroquine there was no change in LC3-II levels in HCE cells grown under hyperoxia, compared to untreated conditions, which might indicate there was no increase in autophagic flux (Fig 4B). On the other hand, we found that further increase in the number of autophagosomes in oxidatively stressed cultures treated with chloroquine compared to untreated condition (Fig 5A), which may be related to increase in the autophagic flux. These findings suggest that the oxidative stress increases the synthesis of autophagy-related membranes occurs simultaneously with low or partial levels of impaired autophagic flux [41, 42]. Hence, augmented LC3-II protein levels were seen in both HCE and HCF cells under oxidative stress, but presence of chloroquine treatment did not alter the levels of LC3-II in normoxic and hyperoxic conditions (Fig 4C). This indicates that the LC3 turnover levels could be similar between the cell types in response to oxidative stress. p62/SQSTM1 has a major role in the association of LC3 with ubiquitinated substrates. In mammals, inhibition of autophagy correlates with increased levels of p62. Similarly, decreased p62 levels are associated with autophagy induction. We found increased levels of p62 in oxidatively stressed HCE cells compared to normoxic conditions. This is an agreement with previous studies on acute myeloid leukemic cells, which showed that upregulated levels of p62 were associated with increased autophagic flux [43]. Though there is no clear correlation between increase in LC3-II and decrease in p62 levels, it is often used to assess the induction of autophagy or impaired autophagic flux. This suggests that p62 can be used in combination with other methods to observe the autophagic flux rate [42].

We hypothesize that an elevated number of autophagosomes (Fig 5A) and turnover levels of LC3-II (Fig 4B) in hyperoxic conditions might induce autophagy along with low levels of autophagic flux as a response to the oxidative damage in HCE cells. Oxidative stress and abnormal autophagy have been implicated in the pathogenesis of other ocular disease conditions as well [35, 44]. Accumulation of autophagosomes and lack of autophagic clearance of mutant-TGFβI protein in corneal fibroblasts of patients with granular corneal dystrophy plays a major role in the pathogenesis [45, 46]. Dysregulation of autophagy as a result of age related oxidative damage has been reported in human trabecular meshwork tissues of healthy donor corneas[47]. Lysosomal dysfunction observed in trabecular meshwork cells under chronic oxidative stress may therefore contribute to the pathogenesis of glaucoma [35, 37]. Another study has demonstrated activation of autophagy mediated cell death during oxidative stress in photoreceptor cell lines (661W) and *in vivo* mice models of retinal disorders [48]. In age-related macular degeneration, increased accumulation of lysosomal lipofuscin and reduced autophagic activity has been implicated in the degeneration of RPE cells due to oxidative damage [49–51]. These reports illustrate a strong association between oxidative damage, autophagy dysregulation and disease progression across several ocular disorders. Interestingly, decreased levels of phosphorylated Akt and p70S6 kinase, an upstream and downstream target of mTOR (mammalian Target of Rapamycin) suggests that autophagy may be regulated through AKT/mTOR mediators in KC corneas and oxidatively damaged HCE cells. Induction of autophagy by a partial inhibition of the mTOR pathway in oxidatively stressed trabecular meshwork cells has a possible role in the pathogenesis of glaucoma [35]. Decreased expression of LC3-II and LAMP1 proteins correlates with levels of phosphorylated Akt/p70S6-kinase in the cone region of KC epithelium across all grades. Hence, we hypothesized that mTOR might be a regulator for the formation of autophagosomes and lysosomes. Our data suggests that the modulation of autophagy might be a potential therapeutic target for KC. Autophagy inducers (trehalose) have been shown to maintain cellular integrity and reduce matrix metalloproteinase activity in corneal epithelium exposed to UV-B radiation [52, 53]. It would be interesting to study the effects of autophagic machinery on a KC *in vitro* model using HCKs, HCFs and HKCs grown in 2D/3D culture system [54]. A limitation of our study is the lack of protein level evidence of autophagic markers, secondary to the minute debrided epithelium that is available, in addition to the tissues being further divided into cone vs periphery samples. Also, fully differentiated corneal epithelial cells (which are not obtained from the limbal region) do not grow in culture, therefore further limiting the options for obtaining more detailed biochemical evidence of autophagic deregulation at the protein level. However, it is possible that analysis of tear levels of autophagic markers from large patient cohorts will help further validate these findings.

In conclusion, we propose that differential expression of autophagy related proteins or impaired autophagy regulation due to oxidative damage in the cornea might be involved in the pathogenesis and progression of KC. Targeting the autophagic lysosomal pathway may offer new therapeutic approaches for the treatment of KC.
